# Engineer the ocean to absorb more carbon: the ocean negative carbon emissions (ONCE) program

**DOI:** 10.1093/nsr/nwae468

**Published:** 2024-12-23

**Authors:** Weijie Zhao, Xiaoling Yu

Humans produce around 420 billion tons of wastewater every year, most of which is discharged into rivers and eventually into the sea. If we alkalize the wastewater before it is discharged, the alkaline water will enhance the ocean's ability to absorb carbon dioxide (CO_2_) from the atmosphere, thus reducing the concentration of atmospheric CO_2_, resisting the greenhouse effect and global warming [1]. This is one of the approaches for mitigation of global warming proposed by the Ocean Negative Carbon Emissions (ONCE) UN international program led by Prof. JIAO Nianzhi (焦念志).

At the United Nations Ocean Decade Conference in April 2024, Jiao gave a speech and talked about the wastewater alkalinity enhancement approach. After his speech, a US scientist came to him and said: ‘Your approach is really effective. We have tried it practically and seen good results.’

‘I was surprised,’ Jiao said in an interview with *NSR*. ‘I didn't expect that this approach would be tried in the United States so fast, even faster than in China.’ Actually, companies in Canada and other countries are also investigating this approach.

In addition to wastewater alkalization, ONCE has proposed many other approaches [2,3]. For example, reducing terrestrial fertilization would improve offshore eutrophication, thereby reducing carbon emissions in estuaries and coastal waters.

In another approach, scientists target on methane, another important greenhouse gas. Currently, methane released by cattle and other ruminants account for nearly one third of human-related methane emissions. It has been shown that if we add ocean-farmed seaweed into cattle feed, microorganisms in the cattle's digestive tract can be effectively regulated and the methane production and emission during the digestive process will be dramatically reduced. Moreover, this approach can reduce cattle's grazing on pasture, thus protecting vegetation and reducing soil erosion.

Furthermore, artificial upwelling can be introduced into the marine pastures to pump the low-temperature, nutrient-rich deep seawater to the upper layer. On the one hand, it can provide and balance the nutrients needed for aquaculture, increasing CO_2_ fixation. On the other hand, the compensatory downwelling caused by the artificial upwelling brings oxygen-rich surface seawater down to the deep layer, relieving the oxygen deficiency there and promoting the sustainability of the ecosystem.

In 2022, the Global ONCE research program proposed by Jiao, as one of 56 current programs of the UN Decade for Ocean Science for Sustainable Development (the Ocean Decade), was formally approved. The program has attracted 79 universities/institutions from 33 countries to join [4]. In 2023, ONCE was approved by the Chinese government as an international big science program.

The International Organization for Standardization (ISO) established the working group named ‘Ocean Negative Carbon Emissions and Carbon Neutrality’ (ISO/TC8/WG15) in 2023, and on November 22nd 2024, the first international standard in this field (number ISO/NP 25283–1) was approved with a 100% voter turnout and 0 dissenting votes.

‘The fact that the Global ONCE program is initiated by Chinese scientists is a major advantage,’ said Dr. Vladimir Ryabinin in an interview with *NSR*. Ryabinin served as the Executive Secretary of the Intergovernmental Oceanographic Commission (IOC) of the United Nations Educational, Scientific and Cultural Organization (UNESCO) for nine years from 2015 to 2023. ‘The Ocean Decade is progressing, however IOC, as its coordinator is noticing that only few Decade activities are led by developing countries and Small Island Developing States. There is a need to expand the number of countries able to conduct cutting-edge climate and ocean research. Global ONCE will make a contribution to making the world more “multilateral”, and this is a way to make the world more peaceful.’

**Figure 1.**
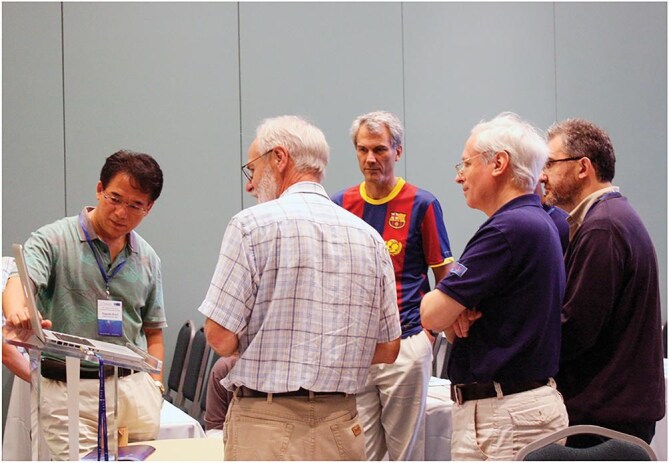
Jiao (left) in discussion with members of the MCP working group of the Scientific Committee on Oceanic Research (SCOR). Image: courtesy of Prof. Jiao.

## MCP: THE FOURTH OCEAN CARBON PUMP

The origin of the ONCE program can be traced back to the early 1990s, when Jiao started his research on ocean new production, which is one of the few quantitative indicators for ocean carbon sequestration. Based on these works, Jiao later described the concept and mechanism of the microbial carbon pump (MCP) [5], and delved into the exploration on how to utilize these carbon pumps to meet the challenges of climate change.

The ocean is the largest active carbon pool on our planet. It is about 20 times that of the terrestrial carbon pool and 50 times that of the atmospheric carbon pool. In the process of carbon entering the ocean from the atmosphere, then depositing into the deep sea from the surface, and finally long-term preservation in the deep sea, four ‘carbon pumps’ play a major role.

The solubility carbon pump (SCP) is the basic ‘physical pump’, that is, CO_2_ is dissolved directly from the atmosphere into the ocean. SCP is directly regulated by the partial pressure of gases in the atmosphere and in the ocean surface.

After being dissolved in the ocean surface, how does the carbon get down to the deep sea and be preserved? In addition to the effect of diffusion and ocean currents, three other carbon pumps, which are all related to biological processes, are involved.

The first is the biological carbon pump (BCP), which is the process by which organisms convert CO_2_ into organic molecules through photosynthesis. But unfortunately, most of the particulate organic carbon (POC) produced by photosynthesis can only sink to about 200 meters below the sea surface, where the POC will be stopped by the pycnocline—a boundary layer in the ocean where the water density increases sharply. Thus, most POC will be finally degraded above the pycnocline and cannot enter the deep sea for long-term preservation.

The second is the carbonate carbon pump (CCP), which refers to the process in which marine organisms use dissolved CO_2_ to synthesize carbonate shells. Following deposition on the seafloor, these carbonates can enter long-term storage there. However, the CCP process actually releases CO_2_ (chemical reaction: Ca^2+^ + 2HCO_3_^-^→ CaCO_3_ + H_2_O + CO_2_), so it is also called a ‘counter pump’.

Finally, the microbial carbon pump (MCP) described by Jiao refers to the biological process in which various marine microorganisms convert labile organic carbon in the ocean into recalcitrant dissolved organic carbon (RDOC) that can hardly be further used or degraded. After conversion into RDOC, carbon can be accumulated and form a huge carbon reservoir that can be maintained for thousands of years.

Scientists have been observing the existence of a huge RDOC reservoir in the deep sea for half a century, but the enigma of its origin had not been solved until Jiao proposed his MCP theories [6].

‘MCP can accumulate carbon continuously and release carbon rapidly under certain conditions, thus forming a two-way regulator of climate. MCP and the other carbon pumps have played an important role in the climate changes during Earth's geological history,’ said Prof. JIAN Zhimin (翦知湣) of Tongji University in an interview with *NSR*. Jian's research interest involves paleoceanography and paleoclimate evolution. He is also one of the leading researchers of the ONCE program, responsible for the construction of observation platforms, and the scientific research related to climate change.

‘My cooperation with Jiao started very early, because the MCP is able to explain some mechanisms of long-term climate evolution,’ Jian said. For example, the slow accumulation and rapid release of carbon by MCP can explain the significant changes of atmospheric CO_2_ concentration during the glacial-interglacial cycles, which is an internal feedback mechanism of the Earth's climate system and has the potential to improve the famous Milankovitch theory for climate evolution.

**Figure 2.**
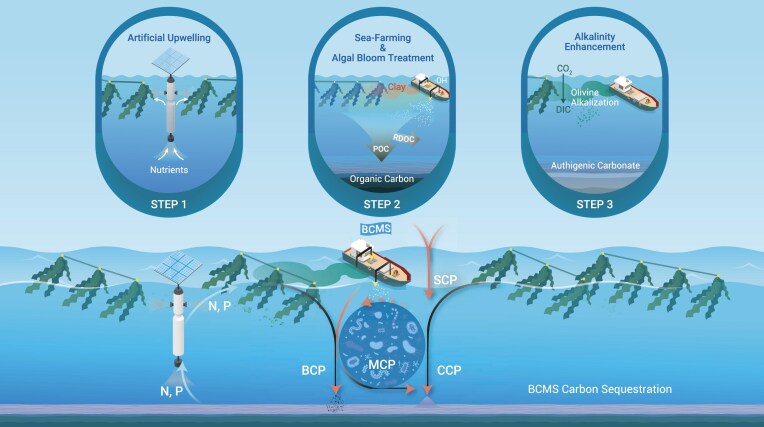
An illustration of the ‘BCP-CCP-MCP-SCP’ (BCMS) ecoengineering approaches. N, P: nitrogen and phosphorus. Image adapted from Ref. [3].

## CHALLENGES OF THE ONCE PROGRAM

Based upon the understanding of the four carbon pumps and their relationships, the ONCE program proposed a series of practical approaches, as introduced at the beginning of this news report.

A major challenge facing ONCE is the related eco-safety concern. ‘During my work for the World Climate Research Programme in early 2000s, an assessment of various geoengineering options was undertaken and it was concluded that potential harm from almost all types of potential geoengineering activities is very likely to exceed the expected benefits,’ said Ryabinin.

Geoengineering activities are actually not allowed in the ocean areas beyond National Jurisdiction, according to both the London Protocol and the newly adopted BBNJ Agreement (The Agreement under the United Nations Convention on the Law of the Sea (UNCLOS) on the Conservation and Sustainable Use of Marine Biological Diversity of Areas beyond National Jurisdiction). That is why the large-scale ion fertilization experiments in the high seas were stopped in 2008.

In the Exclusive Economic Zones (EEZs) of different countries, activities that may have an impact on the high seas need to be assessed and reported first before execution. The ONCE approaches are generally focused on the coastal ocean areas, which are within EEZs and are already deeply influenced by human activities. Jiao believes that based on rigorous scientific evidence and following relevant laws and policies, ‘our approaches in these areas are reasonable and legal.’

Jiao is well-aware of the importance of considering the ecological aftereffects. He said: ‘We will try to avoid unexpected follow-up problems caused by human intervention. Our approaches can be likened to traditional Chinese medicine, which takes into account the patient's health in an all-round way, not just the symptoms at a specific organ.’

To verify the ecological safety and effectiveness of the approaches, they need to be piloted on small ocean areas before being scaled up. In China, Jiaozhou Bay, Bohai Bay and several other sea areas, which are relatively closed and greatly affected by human activities, are all candidates for pilot projects. ‘If we can turn a large bay like the Bohai Bay from a carbon source into a carbon sink, it will be a global benchmark,’ Jiao said.

Here comes the second major challenge of the ONCE program: deployment of the approaches is difficult; it requires close communication and cooperation between scientists, policy makers and policy implementers.

Ryabinin highlighted the importance of co-design: ‘Initiators of a program engage in the design of the program the full scope of stakeholders, from scientists to engineers and eventual end users, and give them equal voices, focusing on the whole value chain of the intended activity.’

Co-design can promote the cooperation of not only the different stakeholders, but also the different countries, by urging the participants to spend a larger share of the funding on internationally coordinated projects, but not the activities of individual nations.

The ONCE program hopes to break the barriers between countries and set an example of international cooperation. At present, ONCE has been joined by 33 countries, and plans to establish scientific hubs in Asia, Europe and the Americas to promote relevant scientific research and geo-engineering practices.

As Jiao said: ‘There are three international languages in the world: sport, art and science.’ No matter how the world pattern changes, international resonance and communication in these three areas will never stop.

## STRIVE FOR A ‘SOFT LANDING’ OF GLOBAL WARMING

‘Global warming is closely related to the increase of atmospheric CO_2_ concentration, but it is not a simple linear relationship.’ Jian said. From the last glacial maximum which was 20 000 years ago, to the pre-industrial year 1840, the atmospheric CO_2_ concentration increased from ∼180 ppm to ∼280 ppm, during which period the average temperature increased by ∼6°C; while from 1840 to the present, the atmospheric CO_2_ concentration increased rapidly from 280 ppm to over 420 ppm, the temperature only increased by ∼1.1°C.

As the quantitative relationship between carbon and temperature is unknown, even if we could estimate how much net carbon emissions can be reduced by the ONCE program, we are still unable to accurately calculate the program's potential contribution to alleviating global warming.

Furthermore, we have to keep in mind that the geoengineering methods trying to fix CO_2_ cannot be an alternative to reducing CO_2_ emissions. The latter is still the main approach to achieve the objective set by the Paris Agreement: to keep the global near-surface air temperature increase by the year 2100 within 2°C, or preferably 1.5°C, in comparison with pre-industrial values.

This objective is at risk, and there is a possibility that in spite of all our efforts, humans will not be able to stop the global warming trend in a short time. But that does not mean all our efforts are useless.

‘We should try our best to prevent climate change from happening too fast, as global warming caused by human activities may lead to extreme weather and climate events that harm society,’ said Jian. Perhaps that is why we urgently need ONCE and other programs. Scientists strive to uncover the mechanisms of climate change and reduce net carbon emissions, in order to buy time for human society to mitigate and adapt to climate warming.

In the history of the Earth, we had experienced the very cold ‘snowball Earth’ period when only the equatorial region was not covered by ice and snow; and we also had the warm but still prosperous periods—in the dinosaur's age more than 100 million years ago, when the atmospheric CO_2_ concentration was as high as 1000 ppm, the average polar temperature was as high as 15°C, the ice sheet disappeared and the sea level rose, but it did not impede the prosperity of dinosaurs.

Utilizing the buffering time won by the ONCE program and all the other scientific efforts to reduce CO_2_ net emissions, humans may be able to make a ‘soft landing’ of the global warming crisis, and continue to inhabit comfortably on our blue planet.
